# Thrombolysis ImPlementation in Stroke (TIPS): evaluating the effectiveness of a strategy to increase the adoption of best evidence practice – protocol for a cluster randomised controlled trial in acute stroke care

**DOI:** 10.1186/1748-5908-9-38

**Published:** 2014-03-25

**Authors:** Christine L Paul, Christopher R Levi, Catherine A D’Este, Mark W Parsons, Christopher F Bladin, Richard I Lindley, John R Attia, Frans Henskens, Erin Lalor, Mark Longworth, Sandy Middleton, Annika Ryan, Erin Kerr, Robert W Sanson-Fisher

**Affiliations:** 1 The University of Newcastle, (UoN) University Drive, Callaghan, NSW 2308, Australia; 2 Hunter Medical Research Institute (HMRI) 1/Kookaburra Circuit, New Lambton Heights, NSW 2305, Australia; 3 Hunter New England Health, Lookout Road, New Lambton Heights, NSW 2305, Australia; 4 National Centre for Epidemiology and Population Health, The Australian National University, Barry Drive, Acton, ACT 0200, Australia; 5 Box Hill Hospital, 16 Arnold Street, Box Hill, VIC 3128, Australia; 6 Westmead Hospital, Hawkesbury Road, Westmead, NSW 2145, Australia; 7 The University of Sydney, City Road, Darlington, NSW 2008, Australia; 8 National Stroke Foundation, Level 7, 461 Bourke Street, Melbourne, VIC 3000, Australia; 9 Stroke Services NSW/ACI Stroke Care Network, Level 4, Sage Building, 67 Albert Avenue, Chatswood, NSW 2067, Australia; 10 Australian Catholic University, 40 Edward Street, North Sydney, NSW 2060, Australia

**Keywords:** Collaborative intervention, Thrombolysis, Acute stroke, Evidence-based practice, Quality improvement, Cluster randomised controlled trial, Multidisciplinary approach

## Abstract

**Background:**

Stroke is a leading cause of death and disability internationally. One of the three effective interventions in the acute phase of stroke care is thrombolytic therapy with tissue plasminogen activator (tPA), if given within 4.5 hours of onset to appropriate cases of ischaemic stroke.

**Objectives:**

To test the effectiveness of a multi-component multidisciplinary collaborative approach compared to usual care as a strategy for increasing thrombolysis rates for all stroke patients at intervention hospitals, while maintaining accepted benchmarks for low rates of intracranial haemorrhage and high rates of functional outcomes for both groups at three months.

**Methods and design:**

A cluster randomised controlled trial of 20 hospitals across 3 Australian states with 2 groups: multi- component multidisciplinary collaborative intervention as the experimental group and usual care as the control group. The intervention is based on behavioural theory and analysis of the steps, roles and barriers relating to rapid assessment for thrombolysis eligibility; it involves a comprehensive range of strategies addressing individual-level and system-level change at each site. The primary outcome is the difference in tPA rates between the two groups post-intervention. The secondary outcome is the proportion of tPA treated patients in both groups with good functional outcomes (modified Rankin Score (mRS <2) and the proportion with intracranial haemorrhage (mRS ≥2), compared to international benchmarks.

**Discussion:**

TIPS will trial a comprehensive, multi-component and multidisciplinary collaborative approach to improving thrombolysis rates at multiple sites. The trial has the potential to identify methods for optimal care which can be implemented for stroke patients during the acute phase. Study findings will include barriers and solutions to effective thrombolysis implementation and trial outcomes will be published whether significant or not.

**Trial registration:**

Australian New Zealand Clinical Trials Registry: ACTRN12613000939796

## Background

### Effective treatments for stroke can provide major reductions in health burden

Stroke is a major cause of death and disability internationally, with over 5 million deaths from stroke worldwide [1]. Stroke accounted for approximately 1 in every 19 deaths in the United States in 2009 [2], with US projections indicating that an additional 4 million people will have had a stroke by 2030 [3], a 22% increase in prevalence from 2013 [4]. The mean cost per person for stroke care in the United States in 2009 was estimated at $6,018 [5]. In 2009, The Australian Bureau of Statistics reports an estimated 381,400 Australians (1.8% of the total population) had suffered a stroke [6], with 35% reporting at least one impairment as a result of that stroke [6] and 62% of these reporting their stroke as their main disabling condition [6]. Stroke was the underlying cause of death for 11,220 Australians in 2010 [6] and is the second leading cause of death and a leading cause of long-term disability [7]. In 2012, the total financial cost of stroke was around $5 billion [8] with an estimated burden of disease cost of $49.3 billion [8]. In 2012, there were nearly 50,000 strokes in Australia, with approximately 12,000 people dying as a result of their stroke [8]. In 2009 to 2010, 35,345 hospitalisations were recorded in Australia with a principal diagnosis of stroke [9] and an average length of stay of 9 days [9]. The majority of strokes (89%) are admitted to hospital [10], and approximately 50% of sufferers are left either deceased or dependent [11].

Improved outcomes for acute stroke can be achieved by: use of aspirin within 48 hours to provide a modest absolute risk reduction of 1% [12]; Stroke Care Units (SCU) [13]; and thrombolytic therapy using intravenous tissue plasminogen activator (tPA) administered to appropriate patients according to guidelines and within 4.5 hours of symptom onset [14,15]. A systematic review of randomised trials of intravenous tPA within 6 hours of acute ischaemic stroke onset found intravenous tPA significantly increased the odds of being alive and independent (modified Rankin Scale of 0 to 2) at 3-month follow-up for those treated within 3 hours of onset [16]. Sustained improvements in functional outcome and health-related quality of life have been identified at up to 18-month follow-up [17]. Treatment benefits for tPA are highest if administered within 3 hours of stroke onset [16,18,19] but can extend beyond the 3-hour window [20], with tPA considered safe for eligible patients up to 4.5 hours after stroke onset [21]. The ‘number needed to treat’ with tPA to prevent death and dependency is between 6 and 14, but may be higher depending on the favourable outcome definition and method of calculation [15,22,23]. Although there has been some debate about the strength of the evidence provided by thrombolysis [24,25], the treatment is endorsed via national and international guidelines [26-29].

### Thrombolysis is a cost-effective stroke treatment when used appropriately

Cost-effectiveness analyses have indicated that high-cost treatments for acute stroke can provide major public health benefits even when minor reductions in disability are achieved [30]. A number of studies have identified thrombolysis as being a cost-effective treatment when used appropriately [31-34]. A UK study estimated that over a lifetime, tPA was associated with cost-savings of £96,565 per quality-adjusted life year [33]. The Model of Resource Utilization, Costs, and Outcomes for Stroke (MORUCOS) study found that tPA saved 155 disability-adjusted life years in a group of 256 eligible patients and concluded that tPA is more effective and cost-saving than treatment with aspirin [32].

### Rates of thrombolysis remain low internationally

Despite evidence for the cost-effectiveness and safety of thrombolysis along with international recommendations being in place for a number of years [21,35,36], there has been little overall growth in thrombolysis rates. Thrombolysis rates have remained relatively low in a number of countries over the past decade: In the US, tPA administration rates increased from 0.87% in 2001 to 2.40% in 2006 [37]. In the UK in 2008, while an estimated 26% of acute stroke patients were eligible for thrombolysis, only 1.4% were administered the treatment [38]. Only 4.3% of ischemic stroke patients in the California Acute Stroke Pilot Registry received thrombolysis [3]. In Australia, despite making major progress in terms of wide establishment of stroke care units, tPA rates are approximately 7% [30].

### Achieving large-scale increases in rates of thrombolysis is challenging

Changing practice is an acknowledged challenge in a number of settings [39]. Improving rates of thrombolysis delivery, even after medical treatment has been sought, involves challenges at several levels, including paramedic recognition of stroke prior to hospital arrival, attendance at a tPA-capable hospital, prompt triaging from the Emergency Department to a Stroke Care Team, rapid access to CT or MRI scan to establish ischaemic stroke, and the availability of appropriately-trained clinicians to provide tPA. A description of the series of tasks involved in the delivery of timely thrombolysis (see Table 1) illustrates the number of tasks, complexity, multidisciplinary nature, and time urgency involved in successful delivery of thrombolysis for acute stroke. An Australian study at a single large teaching hospital (Pre-hospital Acute Stroke Triage [PAST]) developed and implemented a pre-hospital stroke assessment tool for ambulance officers and a pre-hospital notification system following assessment of system barriers [40]. The PAST study demonstrated an increase in tPA rates from 4.7% to 21.4% [40].

**Table 1:** Framework for situational analysis exploring phases of care, tasks, staff roles and time frames

**Phase**	**Task**	**Staff**	**Time frame**
**1. Pre-hospital assessment**	*Assess potential tPA eligibility*	Paramedic	<2 h of onset
	Whether probable stroke or stroke mimic, define time of onset, consider comorbidity/frailty, estimate for survival		
	Deliver to tPA-capable hospital	Paramedic	
	Handover to triage or Acute Stroke Team	to ED/SCU	
**2. Triage**	*Assess tPA eligibility*	ED or SCU	<3.5 h of onset
	Onset time, collateral history, mRS, anticoagulants, seizure, serious or advanced terminal illness, history of intracranial haemorrhage or subarachnoid haemorrhage, major internal surgery in last 21 days, heart attack with IV thrombolysis in last 72 hours, stroke severity assessment with National Institutes of Health Stroke Scale, Glasgow Coma Scale, observations		
	Handover to SCU	to Acute Stroke Team	
**3. Clinical assessment**	*Assess tPA eligibility*	SCU Nurse	
	Onset time certainty, functional independence, comorbidity, current medication, relative and absolute contraindications, NIHSS score, collect blood, notify CT scanning & organise transport, organise ECG		
	Handover to Imaging	to Radiography	
**4. Imaging**	Non- contrast CT scan (or perfusion CT or MRI)	Radiography	
	Handover to SCU	to SCU Nurse	
**5. Final clinical assessment**	*Confirm tPA eligibility*	SCU Medical	
	Review information from phase 1-3, focussed history to check for stroke mimic, onset certainty, premorbid functional independence, medication, contraindications, NIHSS, rapid cardiac and vascular screen, check blood sent to lab, review ECG, review NCCT on console with radiographer and again for degree of ischaemic change & possibility of stroke mimic, discuss scenarios with patient and family		
**6. Preparation & delivery**	*tPA Preparation*	SCU Nurse	<4.5 h of onset
	Check serum glucose level, anticoagulant medication, International Normalized Ratio, Blood Pressure, NIHSS.		
	Treat serum glucose or Blood Pressure if necessary and reassess	SCU Nurse	
	Administer thrombolysis, with amount based on estimated patient weight	SCU Nurse	
**7. Monitor**	Monitor and manage neurological status (NIHSS at 0 h, 1 h, 24 hrs), blood pressure and serum glucose level	SCU/ICU staff	24 h from tPA delivery

Achieving multi-site change in thrombolysis rates for acute stroke poses an even more complex challenge, with very few published trials. One study looked at the benefits and challenges of establishing stroke units [41], and two trials have explored thrombolysis in particular. The US Get-With-The Guidelines stroke project – a national quality improvement program – demonstrated an increase in adherence to guideline recommendations for stroke from a baseline rate for intravenous thrombolytics of 42.09% to 72.84% in the fifth year of the project [42]. The PRomoting ACute Thrombolysis in Ischemic StrokE (PRACTISE) cluster randomised trial in The Netherlands used the Breakthrough approach – a quality improvement methodology using Plan-Do-Study-Act (PDSA) cycles – to change clinical practice [43]. The PRACTISE trial found that a significantly higher proportion of intervention group ischemic stroke patients presenting within four hours of stroke onset were thrombolysed (44.5%) compared to ischemic stroke patients in the control group (39.3%) [43]. However, patients in the intervention group experienced higher post-care dependency [43]. The PRACTISE trial authors suggested a need for a more intense intervention with continuous process measures to achieve more substantial improvements in thrombolysis rates [43]. Studies of the Breakthrough approach in other settings have produced mixed results [44,45], which may suggest the need for additional strategies or a more intensive approach in order to achieve sustained change in complex systems. The INSTINCT cluster randomised controlled trial [46] of 24 acute-care community hospitals in the US used a multilevel, barrier assessment-interactive educational intervention. The intervention included educational elements, assessment of barriers, audit and feedback. Trial results indicated a modest increase in the proportion of admitted stoke patients receiving thrombolysis between pre- and post-intervention periods (1.25% and 2.79% at intervention hospitals, compared to 1.25% and 2.10% at control hospitals using Intention to Treat analysis) but showed non-significant differences between groups. The authors concluded that additional strategies are needed in order to increase acute stroke treatment.

Therefore, a need remains for a rigorous trial of usual approaches versus a comprehensive and intensive strategy for increasing thrombolysis rates in a manner suitable for large scale implementation. The Thrombolysis ImPlementation in Stroke (TIPS) study aims to provide a robust test of a theory and evidence-driven multi-component and multidisciplinary collaborative approach designed to achieve sustained change in complex health systems over multiple sites.

## Aims

The study aims to test, using a cluster randomised controlled trial, whether a multi-component multidisciplinary collaborative approach can:

1. Increase the proportion of all stroke patients receiving thrombolysis at intervention hospitals, compared with control hospitals at follow-up (Primary Outcome);

2. Maintain best-practice benchmarks of 30% of patients achieving 3-month post-stroke modified Rankin scores of 0 to 1 (little or no disability) at both intervention and control sites (Secondary Outcome);

3. Ensure that the adverse event rate for major intracranial haemorrhage (parenchymal haematoma) in thrombolysed patients does not rise above best-practice benchmarks of 6% [19] in either intervention or control hospitals (Secondary Outcome).

## Methods and design

**Figure 1:**
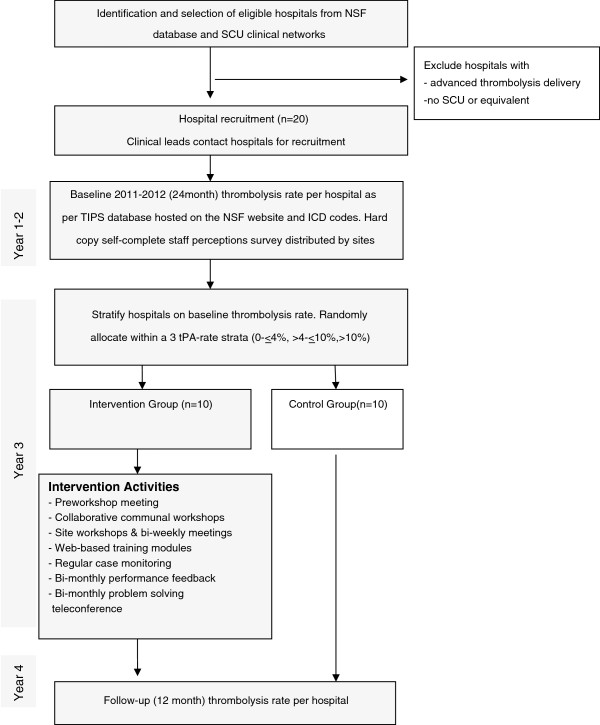
Study design & timeframe.

The cluster randomised controlled trial will involve 20 hospitals in the Eastern Australian states of New South Wales (NSW), Victoria (VIC) and Queensland (QLD). Hospitals are the unit of randomisation and the unit of analysis. SCUs manage the majority of stroke patients across the Eastern states of Australia. Hospitals in the intervention group will receive a multicomponent multidisciplinary collaborative intervention based on behavioural principles as described by the Behaviour Change Wheel framework for the design and implementation of evidence-based practice [47]. The control hospitals will receive no intervention and are free to make practice changes of their own accord. Thus the study is an implementation effectiveness trial rather than an efficacy trial. The follow-up period will be two years post-baseline (*i.e*., Year 1 to 2 = baseline, Year 3 = intervention, Year 4 = follow-up), with an additional three months for measurement of functional outcomes for those thrombolysed in the final month of follow-up. Figure 1 describes the study design.

### Sample

#### Inclusion criteria

Eligible hospitals are those with a Stroke Care Unit or staffing equivalent of a stroke physician and stroke nurse; an Emergency Department and where the hospital is at early stages of thrombolysis implementation. All participating hospitals are required to record every consecutive case of stroke and thrombolysis, including adverse events and patient functional outcomes at three months. Both public and private hospitals are eligible to be included in the sample, as are both teaching and non-teaching hospitals.

##### *Stratified* randomisation

Hospitals will be stratified according to their stroke thrombolysis rates at baseline: very low (0% to ≤4.00%), low (>4.00% to ≤10.00%) or moderate (>10.00%). Baseline thrombolysis rate was considered important for the following reasons: A ‘very low’ thrombolysis rate suggests some impediments to thrombolysis, potentially limiting the ability of the site to respond quickly to the intervention. Sites categorised as having a ‘low’ thrombolysis rate may have the greatest potential for achieving change during the study, due to having substantial room for improvement. Conversely, a site with a ‘moderate’ baseline rate of thrombolysis may have limited opportunity to demonstrate further substantial improvement on the outcome measure.

Hospitals will be randomised at one time (effectively achieving allocation concealment) to either the intervention arm or the control arm with a 1:1 allocation ratio, using a computer generated stratified randomisation scheme in StatsDirect. Intervention hospital staff will not be effectively blinded to their allocation, in that they will be aware of their own involvement in intervention activities. There will be minimal interaction with control hospitals during the intervention phase.

### Procedure

#### Site recruitment

Eligible hospitals were identified from National Stroke Foundation (NSF) audit records and state-based clinical SCU networks. Clinical leaders in stroke at each potentially eligible hospital have been contacted by a clinical leader from the research team to verify eligibility and have been invited in person or by telephone to provide in-principle support to participate in the study following discussions with relevant decision makers at each site. A signed memorandum of understanding or equivalent consent agreement between each hospital and the research team has documented agreement by all relevant parties to collaborate for the whole period of the study. Ethical approval for the study has been obtained from relevant human research ethics committees in each state, from each participating hospital and from The University of Newcastle Human Research Ethics Committee.

#### Data collection

Information on each patient thrombolysed during the study period will be entered into the secure TIPS database hosted on the National Stroke Foundation (NSF) website. Stroke imaging will be entered via an additional INternational Stroke Perfusion Imaging REgistry (INSPIRE) website designed for imaging data that will be linked to the TIPS database. The TIPS database is only accessible via secure logins and will be maintained by the NSF. The patient data will be entered by the stroke care nurse at each participating hospital as de-identified patient unit record data. Data quality including range checks will be undertaken. The research team will not have access to identifying information about individual clinicians, hospital staff, or data identifying individual patients other than in the normal course of their existing clinical duties. Electronic data files will be securely protected so that only the principal researchers will have access to these data. Staff members from participating departments at each hospital will be asked to complete an anonymous baseline voluntary survey regarding perceived barriers and facilitators to thrombolysis implementation at their hospitals. This survey will be repeated at follow-up.

### Measures

#### Primary outcome

Numerator: The numerator is the number of cases treated with thrombolytic therapy in each hospital. The following details will be recorded for each thrombolysed case: age, gender, date and time of stroke onset, date and time of arrival at treating hospital, date and time of brain imaging examination, time of tPA treatment, mRS grade before stroke (premorbid mRS), stroke severity (NIHSS Scale), hypertension, diabetes, hyperlipidaemia, smoking status (current or previous), previous diagnosis of stroke, atrial fibrillation (including paroxysmal), congestive heart failure, aspirin at stroke onset, dipyridamole at stroke onset, clopidogrel at stroke onset, anticoagulants (oral) at stroke onset, glucose level before treatment, and mRS at three months post-treatment. Patient imaging data will be uploaded via the INSPIRE database and will include: non-contrast CT (NCCT), perfusion CT (CTP), CT angiography (CTA), and Magnetic Resonance Imaging (MRI).

Denominator: The denominator is the number of cases with a primary discharge diagnosis code of stroke (ICD-10-AM I60.9, I61, I63, I64) that will be extracted from hospital records. The accuracy of ICD coding for all types of stroke is good [48-51], with a NSW tertiary hospital reporting 96% accuracy in stroke discharge diagnosis identified by a random selection of stroke medical records [50,51]. Another Australian study reported high levels of reliability and adherence to coding standard for ICD-10 codes [52], with a study of stroke attack rates and case fatality in the Hunter Region reporting 97.5% accuracy between stroke coding and audit [53]. Validation of Australian data sets for stroke diagnosis found 93% coding accuracy levels [49], and a Canadian study reported 92% coding accuracy using ICD-10 codes for stroke [54].

The primary outcome is the stroke thrombolysis rate for each hospital, calculated as the number of stroke cases thrombolysed divided by the total number of individuals with a stroke primary discharge diagnosis.

#### Secondary outcome

The secondary outcomes include intracranial haemorrhage as defined by the SITS registry and functional outcomes at three months post stroke using the modified Rankin Scale (mRS) [55]. Intracranial haemorrhage events (parenchymal haematoma) will be identified from the TIPS database and will be monitored annually during the intervention and follow-up phases by the research team to ensure that it does not exceed international benchmarks [56]. Sites will be provided with additional support if benchmarks are exceeded. Monitoring of secondary outcomes will not interfere with established governance processes for reporting and monitoring of adverse events in the health system. Functional outcomes will be scored as good (mRS = 0 to 1) or poor (mRS = 2 to 6) by the treating clinician or stroke nurse. The mRS has demonstrated reliability and validity for use by clinicians and stroke nurses [57]. The percentage of tPA-treated stroke cases having a good outcome (mRS <2) at three months post stroke will also be monitored annually by clinical experts in the research team through the post-intervention NSF data collection.

### Process measures

Pen and paper staff surveys will be conducted at all participating hospitals regarding perceived barriers and facilitators to tPA implementation. Survey items include: perceptions of tPA efficacy, local policies about tPA, knowledge of tPA, skills relating to assessment for and administration of tPA, challenges in tPA implementation, training relating to tPA, expertise and confidence in relation to tPA, contingencies relating to tPA, participant’s gender, age, experience, staff role, and estimated rates of tPA administration. For intervention hospitals, engagement with each intervention activity will be assessed, including number and type of staff from each site attending each workshop or meeting. Assessment of baseline overall readiness for change will also be assessed by a member of the research team using a readiness for change checklist based on the work of Warrick *et al*. (2009), Kasurinen *et al.* (2002), and the National Institute for Health and Clinical Excellence (2007). The full version of the process measure tools can be obtained from the corresponding author.

### *Intervention* components and activities

**Figure 2:**
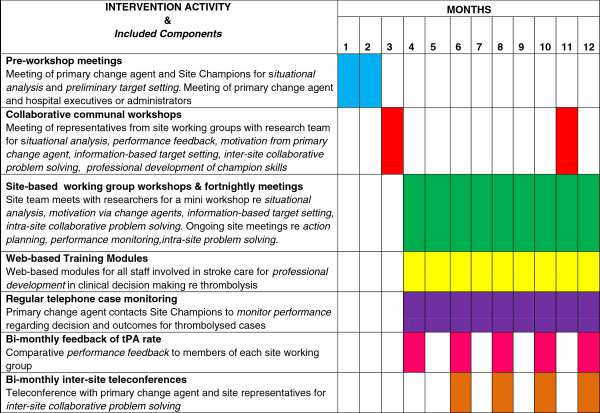
Timing of delivery of intervention activities.

The TIPS intervention components described below are grounded in behavioural theory and accord with the Behaviour Change Wheel (BCW) method [47]. The BCW emphasises the importance of ensuring that proponents have the capability, opportunity and motivation to perform the desired behaviour through interventions such as education, persuasion, incentivisation, coercion, training, restriction, environmental restructuring, modelling and enablement. A situational analysis based on the ‘patient journey’ is a foundational intervention component in that it delineates the steps and associated staff roles involved in successful delivery of thrombolysis. The TIPS intervention components are delivered via a number of intervention activities, the timing and content of which is presented in Figure 2.

## Component 1. Situational analysis – clarifying the patient journey

Careful delineation of the phases of care was carried out by the research team on the basis of earlier work at a single site [40], beginning at the point of seeking emergency medical care (hospital or paramedic). The situational analysis follows the journey of a stroke patient including pre-hospital assessment, triage, clinical assessment, imaging, final clinical assessment, preparation and delivery of thrombolysis. The tasks and staff roles involved along the patient journey are described in Table 1. Examination of how well each of the steps and roles described in the situational analysis will be implemented at a particular site is embedded in a number of intervention activities, including a pre-workshop meeting between the primary change agent and the champions at each site, collaborative workshops involving a team from each site, and site-based working group meetings. In the context of each of these intervention activities, the situational analysis facilitates identification of barriers and facilitators to increasing the number of patients who are appropriately thrombolysed.

## Component 2. Change agents - educating, persuading and modelling

Much of the work on organisational change emphasises the importance of champions or leaders in order to increase the rate of adoption of a desired change [58,59]. In the context of TIPS, change agents will interact with participants to educate, persuade and model desired behaviours throughout the change process. TIPS change agents include primary change agents who liaise with each of the intervention sites and change agents or ‘champions’ located at each site. Stroke nurses from the research team will act as stroke nurse ‘champions’ to monitor and encourage completion of the nurse professional development training (see Component 5). Members of the research team (CL, MP, CB) have been identified as the primary change agents, selected on the basis of meeting criteria for leadership and ability to enact education, persuasion and modelling at a national level [59]. The activities of the primary change agents involve identification of Site Champions and assisting the Site Champions with obtaining endorsement from senior hospital administrators or executives. During the intervention phase, the primary change agent will make regular contact (by email or telephone) to discuss any questions or concerns relating to recent cases of thrombolysis. The primary change agents will also lead education and training aspects of the communal workshops, and attend site workshops. The Site Champions will be the drivers of change at each site, engage with those involved in clinical work flow decisions, and will form a working group comprising key local staff from para-medicine, emergency, stroke care, and imaging. The Site Champions will lead the site working groups in setting goals, identifying actions, and reviewing performance designed to achieve change. The Site Champions are selected on the basis of clinical role (generally lead neurologist and lead stroke nurse).

## Component 3. Information-based target setting – persuasion and incentivisation

Target setting has been established as an important motivator and driver of change on an individual and system level [60]. Information-based target setting involves a process of setting overall targets for appropriate and achievable rates of thrombolysis for each site. Importantly, a series of interim targets or successive approximations will be set during the course of the intervention period.

During the pre-workshop meeting, the Site Champions at each intervention hospital will set a preliminary target for increased tPA administration at that hospital with the guidance of the primary change agent. This target will be reviewed at the first communal workshop in collaboration with members of the site-based working group. Targets are framed based on baseline thrombolysis rates and should be challenging but achievable to create a collective expectation of change and experience of achievement. Interim targets will also be set, based on perceived achievable changes in performance in successive three-month periods throughout the intervention phase.

## Component 4. Collaborative problem solving - education, modelling and enablement

Collaborative problem solving is central to commonly-used quality improvement strategies [61-63] and aims to harness the knowledge, skills and support of a group. Collaborative problem solving will occur within site working groups during their bi-weekly meetings. Bi-monthly teleconferences between the primary change agent and representatives from each of the intervention site-based working groups will explore experiences of all groups in relation to identifying barriers and implementing solutions. An evidence-based process will be used whereby groups attempt to analyse the problem before attempting to search for a viable solution [63].

## Component 5. Professional development – education, training and restriction

Detailed education and training regarding clinical decision making for thrombolysis will be provided via web-based educational modules with a strong emphasis on achieving accepted levels of competency in key skills such as decision making. Web-based approaches provide highly accessible, flexible and effective approaches to learning, particularly when an interactive case-based approach is used [64]. The TIPS website is available to intervention sites via a unique log-in for each participant.

For clinicians, the website contains didactic training modules with a test at the end of each module and a series of case studies where clinicians test their clinical judgement against a consensus-based logic frame for a set of stroke cases. In each case, the clinician is provided with feedback regarding the appropriateness of the decision and links to further reading if required. Clinicians are asked to continue with the case studies until they have achieved an agreed level of competence in decision making. Lead clinicians are asked to ensure that all relevant staff including emergency physicians and registrars complete the training prior to caring for potential stroke patients. The website provides information and training for paramedics in the identification of potential stroke patients. Both the clinician and paramedic training modules were developed by members of the TIPS research team and colleagues.

NET SMART-Junior, a distance-accessible competency-based learning system covering evidence-based management of acute stroke patients, will serve as the educational platform for standardised nursing education in the TIPS trial. The NET SMART system offers rolling entry/completion times supported by self-learning paced programming. NET SMART-Junior is accredited by the Arizona State University, College of Nursing and Healthcare Innovation (Tempe, Arizona, USA) to deliver continuing education credits internationally for completion of modular units consisting of pre-test/post-test and clinical skills measures. TIPS participants will be asked to complete a selected number of training modules and to discuss their results with their nurse ‘champion’.

The TIPS website also contains scientific literature relating to thrombolysis, and tools and resources such as those from the PAST trial [40]. In addition to training in decision making skills, training in the skills of being a champion will be provided. Change champion training will also occur as a component of the communal workshops in the form of group-based reflective activities, followed by self-directed tasks and opportunities for engaging with ongoing external training or self-directed learning.

## Component 6. Performance feedback - persuasion, modelling

Given the strategic importance of providing performance feedback in order to achieve clinical practice change [65,66], local and comparative feedback will be provided. Sites will be provided with their three-monthly estimated proportion of ischaemic stroke cases who receive thrombolysis, graphed against site targets. Comparative data will be provided, showing each site how it compares to other intervention hospitals in a de-identified format (*i.e*., other hospital names not disclosed), to create a positive level of competition among peers. This information will be made available to the participants via their unique logins on the TIPS website and also forwarded directly to Site Champions.

## Intervention sustainability

Achieving sustainability: Throughout the intervention period, there will be a focus on identifying and enacting strategies to embed changes in practice and ensure ongoing practice is monitored via mandatory recording and reporting of thrombolysis delivery. During every collaborative intervention activity (*e.g*., workshops, site-based meetings, inter-site teleconferences), sustainability will be discussed in order that a strong focus on sustainability is maintained. Strategies for achieving sustainability will include: i) key steps in the process of care (*e.g*., time from arrival to handover to stroke team and coverage of imaging services) as part of local standard recording and internal reporting; ii) establishing ongoing communities of practice/clinical networks or including thrombolysis as one of the priority initiatives of existing clinical networks; iii) establishing networks for ongoing mentoring, including formal mentoring relationships that link groups of staff across hospitals; iv) including numbers and proportions of thrombolysed patients in standard reporting to hospital executives and local health districts; v) incorporating the requirement for completion of the TIPS web-based training modules as part of orientation for medical staff in all relevant departments; and vi) establishment of the web-based training as a locally-recognised professional credential required for those involved in the care of stroke patients in the local health district. Site-based working groups will also be encouraged to identify and enact other locally-relevant strategies for achieving sustainability.

Measuring sustainability: Progress in achieving the above sustainability strategies will be monitored during both the intervention and follow-up phases via bi-monthly site teleconferences and post-test staff surveys as part of the study process measures. The degree to which each of the intervention activities (regular site working group meetings, completion of training modules by new staff, entry and monitoring of the number of thrombolysis cases) continues to occur during the follow-up period (Year 4) will also be monitored as part of the study process measures.

## Sample size

From baseline data, it is estimated that participating hospitals will have an average of approximately 150 stroke patients per year, that 5% of stroke patients in the control group will be prescribed tPA, and that the average co-efficient of variation across strata will be approximately 0.4. With 10 hospitals per group, the study will have 80% power with a 5% significance level to detect an absolute difference of 7% to 10% [67]. The subgroup analysis of hospitals with baseline tPA rate of <10% will also have similar power to detect this difference, as, although the number of hospitals per group will be smaller, the baseline tPA rate will also be lower.

## Statistical methods

Primary outcome: Analysis will involve cluster-level summary data. The difference in post-intervention thrombolysis rates between intervention and control groups will be compared via a two-stage approach to adjust for baseline thrombolysis rates, using a stratified *t*-test.

Secondary outcomes: The proportion of thrombolysed cases with good outcomes (mRS scores of 0 to 1) and the proportion with intracranial haemorrhage at each hospital will be compared to benchmarks (using a one tailed hypothesis test) and analogous methods to stopping rules for randomised clinical trials. Thresholds beyond which intervention is required will be calculated using one-sided hypothesis tests adjusted for annual checks. There will be intervention thresholds, beyond which quality assurance processes would be instituted, and stopping thresholds, beyond which tPA administration would be suspended until processes are reviewed. Because we are conducting three analyses, we have used the method of O’Brien-Fleming to determine significance levels for each of the three analyses (this method provides more stringent stopping criteria for early analysis). The appropriate significance levels are 0.0006, 0.0151 and 0.0471 for analysis 1, 2 and 3 respectively. Analysis will be intention to treat in that all individuals administered tPA should be included in the study database, and the denominator will be obtained directly from computerised hospital admissions data.

Secondary analyses: Given that there may be limited potential for hospitals with high baseline tPA rates to substantially further increase tPA administration, we will undertake a subgroup analysis of the primary outcome excluding hospitals with a baseline tPA of more than 10%.

## Trial status

The trial has been registered with the Australian New Zealand Clinical Trials Registry: ACTRN12613000939796 and has obtained a UTN number: U1111-1145-6762.

## Discussion

TIPS is one of the few studies to rigorously trial a comprehensive, multi-component and multidisciplinary collaborative approach to improving thrombolysis rates at multiple sites, and builds substantially on the promising work of the PRACTICE trial [43] and the INSTINCT trial [46], which both emphasised the need for a more intensive approach, such as that proposed for TIPS. The study findings will occur in the context of a range of challenges and limitations as described below. TIPS has the potential to identify methods by which optimal care can be effectively implemented on a large scale for stroke patients during the acute phase. Trial outcomes will be published whether significant or not.

### Key study limitations and challenges

#### Measurement of primary outcome – tPA rates

The measurements of both the numerator and denominator for the primary outcome are challenging. The study numerator is entered by the participating site, rather than by an independent, blinded auditor, therefore introducing potential bias. The study denominator – medical coding of all stroke cases – is independent and blinded in that medical coders at each hospital are unaware of the project. The denominator is also an approximation in that it includes all stroke cases rather than only ischaemic stroke cases.

## Group allocation not able to be blinded

A hospital’s random allocation to intervention or control group cannot be concealed due to the nature of the intervention. Therefore, additional potential bias is introduced.

## Engagement of paramedic/ambulance service

The Ambulance Service has been approached to participate in this project. They will provide the ‘in-field’ context for ischaemic stroke identification and management. It is unlikely but possible that ambulance officers participating in the project will cover both control and intervention hospitals and that any changes in their protocols or procedures resulting from this project may have an impact on both control and intervention sites. This cannot be controlled for in the study design.

## External factors

Other factors that may influence the results of the study, but cannot be controlled for, include policies, guidelines, or any health reforms being rolled out during the intervention period. There may also be new information regarding the tPA drug or other treatments of ischaemic stroke becoming available during the lifespan of this project, thus potentially affecting the results of the study. However, any benefit from these initiatives reasonably can be expected to impact equally on control sites as on intervention sites. Any adverse events associated with this study may also impact the outcomes of the study, especially if they garner media attention. These external factors will be monitored and included in the discussion of study findings.

Notwithstanding these limitations, TIPS has significant rigour given the large cluster RCT design, stratification on baseline tPA rates, and objective outcome measurement.

## Changes to this protocol

The TIPS Working Group will report any changes to this Protocol to the TIPS Steering Committee and relevant Human Research Ethics Committees. Changes will also be reported in published papers emerging from this trial.

## Conclusions

TIPS has the potential to make a major contribution to the implementation of translational literature, given the combination of a theoretically driven, comprehensive intervention strategy and rigorous outcome measurement.

## Abbreviations

TIPS: Thrombolysis ImPlementation in Stroke; tPA: Tissue plasminogen activator (also referred to as PLAT); NSF: National Stroke Foundation (Australia); INSPIRE: INternational Stroke Perfusion Imaging REgistry; SITS: Safe Implementation of Thrombolysis in Stroke registry (Australia); SCU: Stroke care unit; NIHSS: National Institutes of Health Stroke Scale; NHMRC: National Health and Medical Research Council; mRS: Modified rankin score; MORUCOS: The Model Of Resource Utilization, Costs, and Outcomes for Stroke; PAST: Pre-hospital Acute Stroke Triage; CT: Computed Tomography; NCCT: Non-contrast Computed Tomography; CTP: Perfusion Computed Tomography; CTA: Computed Tomography Angiography; MRI: Magnetic resonance imaging; IV: intravenous; ED: Emergency Department; ECG: electrocardiogram.

## Competing interests

The authors declare that they have no competing interests. Boehringer Ingelheim is a collaborative partner in this project and will have a clear interest in increasing the use of the tPA drug. They have, however, no rights to publish any results arising from this project.

## Authors’ contributions

The following authors contributed considerably to study design and critically revised the article: CA, JRA, CFB, HB, GC, CAD, SD, HD, ME, GD, RG, SG, AG, RG, JG, GH, FH, KH, SH, AJ, EK, EL, AL, CRL, RIL, ML, SM, KM, BP, MWP, CLP, MP, CP, SR, AR, RSF, AS, RS. All authors read and approved the final article. In addition to this, CLP drafted the article and assisted with site recruitment, CRL provided clinical expertise and managed site recruitment, CAD and JRA provided statistical expertise, FH provided IT and website expertise, AR co-drafted the article and managed ethics applications and agreements with sites, EK assisted with the acquisition of data, RSF provided health behaviour expertise, MWP, CFB, RIL, EK, ML and SM provided expertise in the development of the TIPS training modules, AG managed ethics applications and agreements with sites, KM assisted with site recruitment and the development of training resources, SR and RS assisted with the development of TIPS training modules, and AS provided health economic expertise. The following authors contributed considerably to the acquisition of data and critically revised the article: IB, CB, EB, ZC, TC, AC, SD, JD, MD, FG, RG, KH, JH, BJ, MJ, MK, MK, MK, SK, PL, SL, KL, BM, EM, KM, KM, SM, EOB, KP, RP, FR, JR, MS, ST, ZT, NT, JW, AW, YW, TW, NW. All authors read and approved the final article. The following authors were part of the writing group for the article: CLP, CRL, CAD, MWP, CFB, RIL, JRA, FH, EL, ML, SM, AR, EK, RSF.

The Thrombolysis ImPlementation in Stroke (TIPS) Study Group:

Mr Craig Anderson^4,6^

Dr Ian Bruce^7^

Dr Heather Buchan^8^

Ms Camelia Burdusel^9^

Associate Professor Ernest Butler^10^

Mr Greg Cadigan^11^

Ms Zoe Campbell^12^

Mr Tim Coles^13 ^

Professor Alistair Corbett^14^

Ms Susan Day^15^

Ms Sonia Denisenko^16^

Ms Jenny Dennett^13^

Associate Professor Helen Dewey^17,18^

Professor Geoffrey Donnan^17,19^

Ms Michelle Doughty^20^

Mr Malcolm Evans^3^

Dr Frances Gearon^21^

Dr Richard Geraghty^22^

Professor Richard Gerraty^12^

Dr Sumitha Gounden^21^

Ms Alice Grady^1,2^

Dr Rohan Grimley^23^

Dr Jeremy Grimshaw^24^

Professor Graeme J Hankey^25,26^

Dr Kelvin Hill^5^

Ms Kim Hoffman^27^

Ms Sue Huckson^28^

Dr James Hughes^29^

Ms Amanda Jayakody^1,2^

Dr Bronwyn Jenkins^30^

Dr Martin Jude^31^

Mr Malcolm Kanard^30^

Dr Matthew Kinchington^32^

Associate Professor Martin Krause^15^

Ms Sarah Kuhle^22^

Dr Paul Laird^33^

Dr Andrew Lee^34,35^

Dr Stanley Levy^36^

Ms Karen Longworth^7^

Ms Beverley Macdonald^36^

Ms Elizabeth Mackey^37^

Dr Krishna Mandaleson^13^

Ms Katherine Mohr^31^

Dr Stephen Moore^27^

Ms Kristy Morris^3^

Ms Elizabeth O'Brien^15^

Mr Bruce Paddock^38^

Ms Kim Parrey^32^

Ms Rachel Peake^29^

Associate Professor Michael Pollack^1,3^

Mr Christopher Price^19^

Ms Shiho Rose^1,2^

Dr Jayantha Rupasinghe^10^

Ms Fiona Ryan^21^

Dr Andrew Searles^1^

 Ms Rochelle Smits^1,2^

Ms Margaret Stevenson^10^

Dr Sanjeev Taneja^20^

Dr Zoe Terpening^39^

Ms Natalie Teasdale^37^

Professor John Watson^39^

Ms Alison Wilson^14^

Dr Yolande Weiner^40^

Associate Professor Tissa Wijeratne^17,37^

Dr Nigel Wolfe^9^

Affiliations

^1^The University of Newcastle (UoN), University Drive, Callaghan NSW 2308

^2^Hunter Medical Research Institute (HMRI) 1/Kookaburra Circuit, New Lambton Heights NSW 2305

^3^Hunter New England Health, Lookout Road, New Lambton NSW 2305

^4^The University of Sydney, City Road Darlington NSW 2008

^5^National Stroke Foundation, Level 7, 461 Bourke Street, Melbourne VIC 3000

^6^The George Institute for Global Health, level 13, 321 Kent Street, Sydney NSW 2000

^7^Coffs Harbour Health Campus, 345 Pacific Highway, Coffs Harbour, NSW 2450

^8^Australian Commission on Safety and Quality in Health Care (ACSQHC), level 5, 255 Elizabeth Street, Sydney NSW 2000

^9^Blacktown Mount Druitt Hospital, 18 Blacktown Road, Blacktown NSW 2148

^10^Frankston Hospital, 2 Hastings Rd, Frankston VIC 3199

^11^Queensland Health Statewide Stroke Clinical Network (SSCN), C/-Clinical Access & Redesign Unit, GPO Box 48, Brisbane QLD 4001

^12^Epworth HealthCare, 89 Bridge Road, Richmond VIC 3121

^13^Central Gippsland Health Service, 155 Guthridge Parade, Sale VIC 3850

^14^Concord Repatriation General Hospital, Hospital Road, Concord NSW 2139

^15^Royal North Shore Hospital, Northern Clinical School, University of Sydney, Kolling Building Level 7, Royal North Shore Hospital, Reserve Road, St Leonards, NSW 2065

^16^Victorian Stroke Clinical Network, 50 Lonsdale Street, Melbourne VIC 3000 Australia

^17^The University of Melbourne, 1-100 Grattan Street, Parkville VIC 3010

^18^Austin Health, 145 Studley Rd, Heidelberg VIC 3084

^19^The Florey Institute of Neuroscience and Mental health, 30 Royal Parade, Parkville VIC 3052

^20^Wollongong Hospital, 1 Crown Street, Wollongong NSW 2500

^21^Orange Base Hospital, Dalton Street, Orange NSW 2800

^22^Redcliffe Hospital, Anzac Avenue, Redcliffe QLD 2040

^23^The University of Queensland, Brisbane, St Lucia QLD 4072

^24^Institute of Population Health, University of Ottawa, 550 Cumberland St, Ottawa, ON K1N 6N5, Canada

^25^School of Medicine and Pharmacology, The University of Western Australia, Nedlands, WA 6009

^26^Department of Neurology, Sir Charles Gairdner Hospital, Hospital Avenue, Perth, WA 6009

^27^Lismore Base Hospital, 60 Uralba Street, Lismore NSW 2480

^28^National Health and Medical Research Council (NHMRC), level 1, 16 Marcus Clarke Street, Canberra, ACT 2601

^29^Tamworth Hospital, 31 Dean Street, Tamworth NSW 2340

^30^Hornsby-Ku-ring-gai Hospital, Palmerston Rd, Hornsby NSW 2077

^31^Wagga Wagga Base Hospital, Docker Street, Wagga Wagga NSW 2650

^32^Port Macquarie Base Hospital, Wrights Road, Port Macquarie NSW 2444

^33^Rockhampton Hospital, Canning Street, Rockhampton QLD 4700

^34^Flinders Medical Centre, Flinders Dr, Bedford Park, SA 5042

^35^Flinders University, Sturt Road, Bedford Park, SA 5042

^36^Campbelltown Hospital, Therry Road, Campbelltown NSW 2560

^37^Western Hospital, Gordon Street, Footscray, VIC 3011

^38^Ambulance Service of NSW, Balmain Road, Rozelle NSW 2039

^39^Sydney Adventist Hospital, 185 Fox Valley Road, Wahroonga NSW 2076

^40^Logan Hospital, Corner of Loganlea Road & Armstrong Road, Meadowbrook QLD 4131

## Roles and responsibilities

The TIPS Steering Committee is comprised of Chief Investigators and external advisors. They are responsible for overseeing the design and milestones of the project. The TIPS Working Group is a smaller group of Chief Investigators and people driving the project, including Research Assistants. They are responsible for data monitoring and report to the TIPS Steering Committee. Boehringer Ingelheim has no representation on either of these committees to ensure that study findings are independent of the sponsor and their competing interest.
